# Editorial: Immuno-Metabolism in Tumor Microenvironment

**DOI:** 10.3389/fimmu.2017.00374

**Published:** 2017-04-05

**Authors:** Heriberto Prado-García, Francisco Javier Sánchez-García

**Affiliations:** ^1^Chronic-Degenerative Department, National Institute of Respiratory Diseases “Ismael Cosio Villegas”, Mexico City, Mexico; ^2^Laboratorio de Inmunorregulación, Departamento de Inmunología, Escuela Nacional de Ciencias Biológicas, Instituto Politécnico Nacional, Mexico City, Mexico

**Keywords:** tumor microenvironment, metabolic reprogramming, metabolic symbiosis, immune suppression, immuno-metabolism

**Editorial on the Research Topic**

**Immuno-Metabolism in Tumor Microenvironment**

From Hanahan and Weinberg’s original description of six hallmarks of cancer (self-sufficiency in growth signals, insensitivity to growth-inhibitory signals, evasion of apoptosis, limitless replicative potential, sustained angiogenesis, and tissue invasion and metastasis) (1), it took only 11 years to add in 2011, based on an increasing body of research, two “emerging hallmarks of cancer”: deregulating cellular energetics and avoiding immune destruction (2). Hanahan and Weinberg also pointed out that, besides cancer cells, tumors contain a repertoire of recruited normal cells that contribute to create the “tumor microenvironment” (2). Deregulating cellular energetics is perhaps most commonly known as metabolic reprogramming, a process that can take place in tumor cells (3), as well as in tumor-infiltrating immune cells (4). The interplay between specific metabolic pathways, in the context of cell maturation, activation, differentiation, and effector functions of immune cells, is currently referred to as immuno-metabolism (5). Metabolic reprogramming and avoiding immune destruction are closely interrelated, since metabolic intermediates from tumor cells may suppress the immune response (4).

The research topic “Immuno-metabolism in tumor microenvironment,” here presented, covers several aspects of tumor and immune cell metabolism, as well as the cell–cell interplay within the tumor, at the metabolic level. In addition, some metabolic-oriented cancer therapies are reviewed (Molon et al.; Ohta; Romero-Garcia et al.; Salazar-Ramiro et al.; Zhang and Ertl).

## Tumor Structure

Tumors comprised an intricate network of tumor cells, fibroblasts, endothelial cells, immune inflammatory cells, adipocytes, extracellular matrix molecules, and soluble factors. All these elements contribute to form a structure that favors the development of gradients of oxygen tension and nutrients availability. According to Zhang and Ertl, it is estimated that 50–60% of solid tumors possesses unevenly distributed areas of hypoxia with consequences for individual cell metabolism, functionality and survival and, finally, for tumor outcome.

## Tumor Cell Metabolism

Tumor cells undergo metabolic reprogramming, which allows for their survival; and it is stromal cells (immune cells, fibroblasts, endothelial cells, etc.) that have to endure the consequences of tumor metabolic reprogramming. Otto Warburg initially described that tumor cells are highly glycolytic, even in the presence of oxygen, and produce large amounts of lactate, up to 40 times as much as that of normal cells. As Romero-Garcia et al. pointed out, lactate is not just a by-product of glycolysis; instead, it is an active metabolite that promotes angiogenesis and induces profound effects on both tumor cells (metabolic symbiosis) and immune system cells (immune suppression). Besides lactate, other metabolites are released within the tumor microenvironment; importantly, adenosine, which accumulates at the tumor extracellular milieu, under hypoxic conditions. Akio Ohta focuses his review on the effects of this metabolite on tumor and immune cells. Adenosine may support tumor progression directly, whereas it suppresses pro-inflammatory activities. Blocking of the hypoxia–adenosine pathway is discussed as a possible target for cancer immunotherapy (Ohta). Interestingly, from these two reviews, it emerges that lactate and adenosine have similar effects on tumors (tumor progression and immune suppression), albeit different mechanisms.

As a result of the increased metabolic activity in the tumor cells, reactive oxygen species are released into the microenvironment, which might favor inflammation and progression of cancer (6). Salazar-Ramiro et al. discuss the imbalance in the redox status in glioblastoma multiforme, and the effects that a highly oxidant environment has on genomic instability, gliomagenesis, and tumor progression and, finally, how the redox balance might be an attractive target for therapy against glioblastoma multiforme.

## Metabolism of Tumor-Infiltrating Immune Cells

Tumor-infiltrating lymphocytes are challenged with a hostile environment, notably hypoxia and nutrients scarceness, not to mention high amounts of lactate, adenosine, and anti-inflammatory cytokines. Tumor areas with low oxygen tension arise from structural and functional abnormalities of the tumor microvasculature and a high proliferation rate of tumor cells. Thus, immune cells are frequently infiltrating hypoxic areas, which might favor the inhibition of immune cell functions. Therapies, such as adoptive T cell transfer or immunomodulatory antibodies should take into account that activated effector T cells will be under hypoxic conditions, which might explain why these therapies have not been as successful as expected. Zhang and Ertl review how hypoxia favors the expression of co-inhibitory molecules, and inhibits cell proliferation and production of granzymes and perforin by CD8+ T cells. The effect of glucose deprivation on T cell activation is also reviewed (Zhang and Ertl). Amino acid metabolism represents an important process that might favor tumor progression and limit immune responses. Molon et al. review the effects of tumor microenvironment in T cell metabolism and the metabolic adaptations of T cells within the tumor, examining some immune checkpoints, such as programmed death receptor-1 (PD-1) and cytotoxic T-lymphocyte associated antigen-4 (CTLA-4), and signaling pathways, such as PI3K/Akt/mTOR, and their role in energetic adaptations of both cancer and immune cells.

## Concluding Remarks

What happens to individual cells within a solid tumor is reminiscent of the concept created by Spanish philosopher José Ortega y Gasset: “I am I and my circumstance; and, if I do not save it, I do not save myself.” In this case, the life of an organism (tissue or cells) is formed by the organism itself and by its medium; if the medium changes the organism changes and *vice versa*. In the original quote, there is not determinism at all, since human beings have *logos*.

If tumor cells are within a hypoxic area, they produce adenosine and lactate, among other metabolites. If in an oxygenated area, tumor cells, that are suited to metabolize glucose by glycolysis, rely on lactate instead, sparing glucose for hypoxic tumor cells. Cytotoxic T lymphocytes and other tumor-infiltrating cells live starved and asphyxiated and yet they fight tumor cells, while their surrounding medium provide immunosuppressive signals that later affect their function.

The five papers that comprise this topic suggest that, perhaps, for effective anti-cancer therapeutics, researches have to consider not only the cells (tumor and immune cells) but the cells and their circumstance, as a whole.

## Author Contributions

HP-G and FJS-G equally contributed to manuscript writing and approved the final manuscript.

## Conflict of Interest Statement

The authors declare that the research was conducted in the absence of any commercial or financial relationships that could be construed as a potential conflict of interest.
